# Direct Identification of Label-Free Gram-Negative Bacteria with Bioreceptor-Free Concentric Interdigitated Electrodes

**DOI:** 10.3390/bios13020179

**Published:** 2023-01-23

**Authors:** Mazin Zamzami, Samer Alamoudi, Abrar Ahmad, Hani Choudhry, Mohammad Imran Khan, Salman Hosawi, Gulam Rabbani, El-Sayed Shalaan, Bassim Arkook

**Affiliations:** 1 Department of Biochemistry, Faculty of Science, King Abdulaziz University, Jeddah 21589, Saudi Arabia; 2 Center for Artificial Intelligence in Precision Medicines, King Abdulaziz University, Jeddah 21589, Saudi Arabia; 3 Department of Medical Biotechnology, Yeungnam University, 280 Daehak-ro, Gyeongsan 38541, Gyeongbuk, Republic of Korea; 4 Department of Physics, Faculty of Science, King Abdulaziz University, Jeddah 21589, Saudi Arabia; 5 Department of Physics and Astronomy, University of California, Riverside, CA 92521, USA

**Keywords:** electrochemical biosensors, bioreceptor-free, biorecognition-element-free, concentric interdigitated electrodes, label-free, Gram-negative bacteria, *Escherichia coli*

## Abstract

This investigation demonstrates an electrochemical method for directly identifying unlabeled Gram-negative bacteria without other additives or labeling agents. After incubation, the bacterial cell surface is linked to the interdigitated electrode through electroadsorption. Next, these cells are exposed to a potential difference between the two electrodes. The design geometry of an electrode has a significant effect on the electrochemical detection of Gram-negative bacteria. Therefore, electrode design geometry is a crucial factor that needs to be considered when designing electrodes for electrochemical sensing. They provide the area for the reaction and are responsible for transferring electrons from one electrode to another. This work aims to study the available design in the commercial market to determine the most suitable electrode geometry with a high detection sensitivity that can be used to identify and quantify bacterial cells in normal saline solutions. To work on detecting bacterial cells without the biorecognition element, we have to consider the microelectrode’s design, which makes it very susceptible to bacteria size. The concentration–dilution technique measures the effect of the concentration on label-free Gram-negative bacteria in a normal saline solution without needing bio-recognized elements for a fast screening evaluation. This method’s limit of detection (LOD) cannot measure concentrations less than 10${}^{2}$ CFU/mL and cannot distinguish between live and dead cells. Nevertheless, this approach exhibited excellent detection performance under optimal experimental conditions and took only a few hours.

## 1. Introduction

Pathogens are organisms that cause disease in humans. They threaten human health, and the number of these pathogens is expanding [1]. The main types of pathogens are bacteria, viruses, and parasites. Bacteria and viruses are present on all surfaces and in the air. Therefore, they can easily be transferred to the hands of an individual who touches an infected surface or object. In that case, they have a good chance of contracting the infection themselves—one of the most typical pathogens is *Escherichia coli* (*E. coli*) [2,3,4]. Therefore, rapid and sensitive detection of bacterial pathogens is a strong demand and is needed for today’s global food market and healthy communities. Furthermore, the required technology can be adjusted for various target organisms.

The traditional techniques used to quantify pathogens in cell culture are time-consuming and require skilled operators to perform the sample preparations. In addition, the operator must be well-trained, experienced, and knowledgeable [5,6,7]. Pathogens can be recognized through yielded antibodies that can naturally occur in an organism, which may usually be present during and after infection and help fight against the disease. When biorecognition elements (e.g., antibodies) are functional for the pathogen, they can immediately detect it. The sensitivity of DNA-based assays is greater than that of antibody-based techniques because it does not rely on capturing a specific protein from the cell surface, which is given for more accurate outcomes [8,9,10,11].

A real-time polymerase chain reaction (real-time PCR) is a technique for replicating DNA molecules using the enzyme DNA polymerase and a set of short oligonucleotide primers. It is used in molecular biology to replicate segments of DNA as templates for further use, such as sequencing, cloning, or producing large amounts of a specific gene product. This technique can detect DNA sequences from multiple organisms simultaneously but does not measure viability or other parameters such as colony-forming units (CFUs). Furthermore, Loop-Mediated Isothermal Amplification (LAMP) is a rapid, sensitive, and specific assay for detecting DNA or RNA from infectious agents. LAMP does not require a thermal cycler and can be performed at room temperature. It is a probationary acute nucleotides-permeable polymerase chain reaction (PCR) that does not need a thermal cycle [12].

The following two methods are used in detecting pathogens. (1) Antibodies are proteins created by the body to help fight off foreign invaders such as bacteria and viruses. They are created in the immune system and react with specific antigens, and they can be used to identify a pathogen. (2) Pathogens can be caught depending on their genes, which are the parts of the pathogen that allow it to survive and reproduce. The genes of a pathogen can be detected using PCR, which copies DNA sequences of a given gene and then amplifies them so they can be identified [13].

Biorecognition elements can be classified into three categories: antibodies, aptamers, and imprinted polymers. However, biorecognition elements can bind with other molecules or cells to detect specific biological features [10,14]. However, biosensors are a new and promising technology in the field of science that deals with diagnosing, identifying, and studying any pathogen. They complement other technologies, including PCR and enzyme-linked immunosorbent assay (ELISA) [15]. The biosensor is composed of two parts: (1) a sensing probe that binds to the target molecule (e.g., a pathogen) and (2) a surface that recognizes the binding event (e.g., an electrode). Furthermore, the biorecognition elements can be immobilized on the biosensor’s surface [16,17,18,19,20]. The current research on label-free biosensors can detect pathogens without labels or tags, making it a promising technology for a diagnostic test performed at the point-of-care.

Pathogen detection methods have limitations; for example, some ways can only see some pathogens or take a long time to produce results. Other methods require expensive equipment or trained personnel. However, it is worth noting that biosensors are compatible with label-free protocols. This means there is no need to label the samples or use fluorescent probes. In addition, label-free biosensors have many advantages over traditional label-based sensors. For example, they are more sensitive, do not require an external energy source, and can detect multiple targets simultaneously. In addition, label-free sensors are considered more accurate than label-based ones because they do not require labels. The former is more commonly used in research, while the latter is often used for clinical applications [21]. The use of a transducer for pathogen biosensing has been investigated for many years. Different transducers, including mechanical and optical, have been used as cantilever biosensors to detect and measure biomolecules, such as nucleic acids (DNA or RNA) and proteins [22]. The device consists of a tiny cantilever usually made from silicon or quartz. When an analyte binds to the surface plasmon resonance (SPR) sensor chip at the end of the cantilever, it causes a change in its physical properties [23].

Furthermore, electrochemical biosensors are devices that use electricity to measure the concentration of a chemical substance in a liquid. They employ conducting materials as transducers, typically platinum or gold electrodes. Additionally, a bio-recognition element can be secured to an electrode so it can be detected electrically. This means that when a pathogen binds to an electrode, there is a measurable change in the current generated by that electrode. This change in current can be used as a signal for an alarm or a trigger for other types of response mechanisms [9,24,25].

Generally, a biosensor detects performance by calculating the linear range (LR), sensitivity, response time, limit of detection (LOD), selectivity, stability, and repeatability. The linear range is the range of concentrations that the sensor can detect. Sensitivity is how much signal is generated per unit of analyte concentration. Response time is the time it takes for the sensor to respond to an analyte concentration change. LOD is the minimum concentration detected with a given signal-to-noise ratio. Selectivity is how well a sensor responds to one analyte over another. Finally, stability refers to how stable a sensor’s response would be in terms of time and temperature changes. Ultimately, repeatability is determined by calculating correlations between repeated measurements taken on different days or times [26].

The demand for innovative biorecognition elements that improve detection sensitivity, which makes it possible to detect low concentrations of a substance in complex samples, is still high. However, aptamers are an example of an innovative biorecognition element that has shown improved selectivity and affinity. Aptamers are artificially created nucleic acids that can be used as biosensors. They can be designed to bind to specific molecules, such as proteins or nucleic acids, with high specificity. One advantage of using aptamers as biosensors is that they do not need to be modified chemically to bind their target molecules [27]. One of the various classes of electrodes, screen-printed electrodes (SPEs) is an electrode created by printing an electrically conductive ink on a substrate such as paper or plastic film using an inkjet printer; they are commonly used in biosensors because they allow for ease of fabrication, low cost, and good sensing properties [28]. The patterned electrodes act as an electrochemical sensor that detects changes in a molecule’s oxidation state or reduction state at its surface by measuring changes in its current–voltage response. The screen-printed electrode can be made with various materials, including gold, platinum, palladium, nickel, carbon nanotubes, and others. The current study addresses the possibility of detecting and evaluating bacterial activity without the required biorecognition element through a concentration dilution approach [29,30,31,32].

One of the optimistic areas of study has been developing label-free biosensor technology expected to revolutionize how we detect pathogens. These authors were working on a new type of biosensor called a microcavity resonator. This device can detect the presence of pathogens in less than 10 s. The device is about one centimeter long and can be placed on a surface to detect pathogens. The device can also detect multiple pathogens simultaneously, unlike current devices, which only work with one type of pathogen at a time [33]. In a recent study, a biorecognition element-free interdigitated microelectrode showed that the parallel electrode design could determine *E. coli* with a 30 μm gap employing an impedance analyzer, and the detection limit was 10${}^{3.2}$ CFU/mL [34]. In a different investigation, machine learning was proposed to improve the implementation of bioreceptor-free biosensors, substituting the bioreceptor with modeling to gain specificity [35]. All these studies have encouraged interest in learning more to theoretically and experimentally pave the way for biosensors without biorecognition elements.

Gold electrodes in sensors are a well-known method that has been used for decades. It relies on the fact that gold is a noble metal, which means it does not react with any of the analytes in the solution. The electrodes are interdigitated, meaning they are both in contact with the solution, and this allows for an efficient charge transfer from one electrode to the other. The Interdigitated Electrode (IDE) is a novel electrode design introduced in the early 1990s by Metrohm. In this work, we will look at how these electrodes work and how the change in electrode design leads to the difference in susceptibility of the signal coming from the label-free Gram-negative bacteria. However, the Gram stain is a differential staining technique used in microbiology to classify bacterial cells as either Gram-positive or Gram-negative. Therefore, Gram-negative bacteria appear blue or purple because of the counterstain crystal violet, while Gram-positive bacteria appear red or pink because of the counterstain safranin. The colorimetric test can distinguish between the two types of bacteria regarding their ability to react with an acidified alcohol solution or a dilute carbolic acid solution [36]. The sensors employed in this work are label-free because they do not require additional chemicals or labels to be added to the sample solution. These sensors have increased steadily over recent years because they offer many benefits over other technologies, such as immunoassays or ELISA tests [37]. Furthermore, this design of IDEs has some advantages that make it more effective than other types of biosensors: (1) smaller size—the interdigitated electrode is much smaller than different designs, which means it can be used on smaller objects such as bacteria in food and water samples; (2) lower cost—the cost of producing an interdigitated electrode is lower than that of other designs because it does not require as much material; (3) more accuracy and sensitivity—the interdigitated electrode can detect a better range of bacteria.

## 2. Materials and Methods

### 2.1. Preparation and Growth of Bacterial Cultures

The Gram-negative *Escherichia coli* (ATCC 11775) was obtained from the American Type Culture Collection. The bacteria is a member of the Enterobacteriaceae family, and this organism is used as a model for Gram-negative bacteria. It is a rod-shaped, facultative anaerobe found in the environment and human and animal feces. It was cultured for 24 h at 37 °C in a nutrient broth—REF: M002-500G (HiMedia Laboratories Private Limited, Mumbai, India). The nutrient broth is a type of liquid that contains all the necessary nutrients for the growth of the *Escherichia coli* bacterium. The bacteria were then transferred to a Petri dish with agar, and the resultant bacterial colony was observed. One CFU is one bacterial cell that can form colonies on agar plates.

*Escherichia coli* cells are serially diluted in 0.9% *w*/*v* normal saline solution. Detection is conducted by taking a sample of the solution and measuring it with Interdigitated Electrodes (IDEs), which were used to observe the effects of different concentrations of the *Escherichia coli* cells. First, viable cells were enumerated by the plate count method. The enumeration method used to count cells using a plate count technique can be either viable or non-viable, depending on the type of cells being measured. The Quebec colony counter counts the number of colonies in a Petri dish. The counter consists of a counting chamber and a counting grid. The counting chamber is filled with a suspension of bacteria, and the grid is placed on top of it. A single colony will form one spot on the grid, which can be counted by looking at the grid through an eyepiece. Serial dilutions were then made at the desired cell densities (10${}^{-1}$ to 10${}^{-7}$) starting from 126 × 10${}^{7}$ CFU/ml. Finally, 10 μL of each dilution was pipetted and spread on the biosensor’s sensing area (i.e., the Interdigitated Electrodes). The bacterial concentrations were confirmed before the measurement and modified to 10${}^{7}$, 10${}^{6}$, 10${}^{5}$, 10${}^{4}$, 10${}^{3}$, 10${}^{2}$, and 10${}^{1}$ CFU/mL.

### 2.2. Electrochemical Technique

Cyclic voltammetry (CV) is employed to study how electrochemical reactions occur in analyte solution (bacterial cells—*Escherichia coli*—suspended in normal saline). It uses an electrochemical detector to measure the potential difference between two electrodes and the analyte at different concentrations. CV measurements were used to study the electrochemical properties of Gram-negative bacteria at different scan rates in normal saline solution with inoculating *E. coli*.

The saline solution acts as a buffer, protecting the bacteria from sudden changes in pH or temperature that can cause damage or death. However, normal saline contains a substance whose oxidation and reduction (redox) potential are measured, and other substances, such as bacterial cells, that may affect its redox properties. Next, a voltage is applied across the electrodes by connecting them through an electric power supply, and this causes a current to flow between them. This electric potential difference produces an electric field across the solution that can be detected by measuring its change in current flow during a time interval. The measurements were performed with an Autolab PGSTAT302N potentiostat/galvanostat (Metrohm AG, Switzerland) connected to a personal computer. The Nova ver. 2.1.5 electrochemical software was used to collect and analyze data in this experiment.

### 2.3. Microelectrode Sensors

The three types of sensors in this work were purchased from Metrohm DropSens (Oviedo, Spain). Their electrode design is based on well-known and widely used electrochemical biosensors, which use microelectrodes with different geometries to study their effect on biomolecules. The electrodes are fabricated using conventional photolithographic techniques, which allows for high throughput fabrication at a low cost. The shape and size of an electrode can affect its ability to generate current and how quickly it will corrode. Electrodes with sharp edges typically corrode more rapidly than those with smooth edges. These specific electrode designs are often used for detecting and quantifying bacteria because they provide a large surface area with good electrical properties. For instance, the narrow gap between two electrode surfaces was more effective at generating current than one with a wide gap.

#### 2.3.1. G-MEAB222

The first design was G-MEAB222, a microarray-electrode comprising three electrodes—a counter electrode, a reference electrode, and a working electrode—all made of gold on a glass substrate. It can determine the electric potential difference (voltage) across a membrane, ion channel, or molecule of interest. The working electrode is the one that generates the current that is coupled to the counter and reference electrodes. This design of microarray-electrode can be used for the electrochemical properties of individual molecules or bacterial cells. For example, it has been used to measure the electron transfer rates between individual molecules in contact with one another. The G-MEAB222 is still being studied for its usefulness in measuring cell membrane potentials and ionic currents in cells. The electrode surface has been micro-perforated in 21 bands of 10 μm width and distance between bands of 100 μm to achieve steady-state currents with redox systems.

#### 2.3.2. G-IDECONAU10

The second design was G-IDECONAU10 InterDigitated Concentric Gold Electrodes, composed of two interdigitated electrodes (IDEs) with two connection tracks, all made of gold, on a glass substrate. The interdigitated electrodes’ arrangement in concentric circles design enhances sensitivity and detection limits. Therefore, decentralized assays are suitable for developing specific (bio)sensors and other electrochemical studies. This electrode’s dimension of bands/gaps is 10 μm. A novel and creative design are used to measure solution conductivity and produce high-quality results. The electric field lines in concentric electrodes are curved, and their shape allows for higher voltages to be applied. As the voltage increases, the electric field lines will be close to each other, and the charge density will increase.

#### 2.3.3. G-IDEAU10

**Figure 1:**
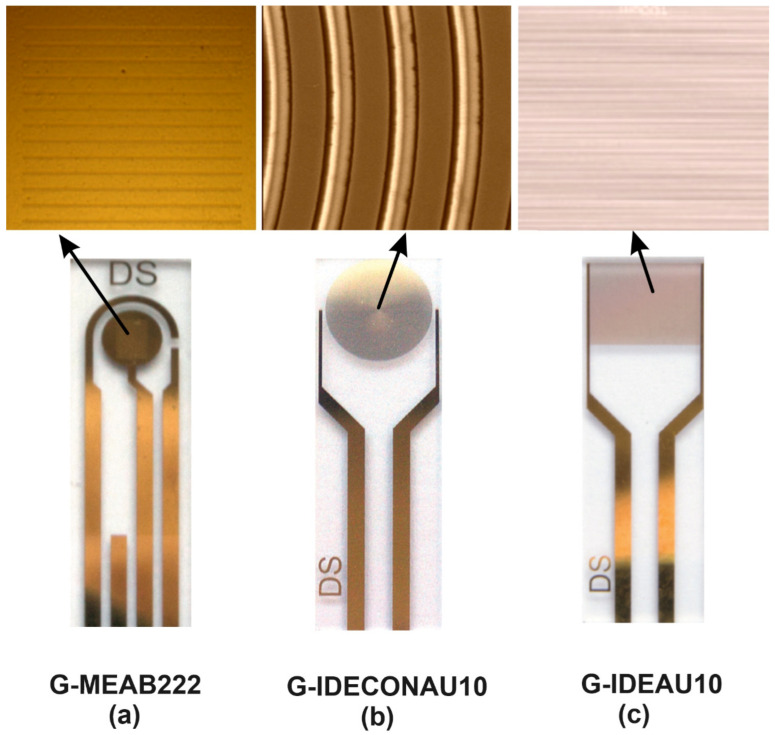
The three different electrodes were used for biosensing applications. From left to right: (**a**) G-MEAB222, the top photo shows the optical microscopy image of the design of the microarray electrode, and the bottom one shows the whole picture of the sensor [38]. (**b**) G-IDECONAU10, the top photo shows the scanning electron microscopy (SEM) image of the design of the concentric circles, and the bottom one shows the whole picture of the sensor [39]. (**c**) G-IDEAU10, the top photo shows the optical microscopy image of the capacitor array design, and the bottom one shows the whole sensor picture [40].

The final design G-IDEAU10 electrode comprises two interdigitated electrodes with two connection tracks of a capacitor array made of gold on a glass substrate. 10 μm separates the electrodes. This can measure the voltage change between the two electrodes due to changes in bacterial membrane potentials caused by ionic currents flowing through bacterial cells. Figure 1 shows the electrodes used and their designs. The electric field lines in a parallel electrode are straight.

A 10 μL capacity sample is sufficiently restricted by foam insulation. This foam insulation has a high thermal resistance and can withstand high pressures. The electrodes were used for holding the bacterial culture. Every sensor was rinsed with 70% isopropyl alcohol and sterile deionized (DI) water and allowed to dry for one day, and then the test solution was applied. The sensors were often disposed of after one use. Before taking any CV measurements, the bacteria were incubated for several minutes on the biosensor, and it was found that they did not grow well on it.

## 3. Results and Discussion

This work found that using gold concentric electrodes without biorecognition elements effectively identified Gram-negative bacteria. Furthermore, the results showed that the electrochemical method was accurate and could be used as a practical solution for identifying Gram-negative bacteria.

The sensitivity and specificity of biosensors are essential properties and the main challenges in designing a biosensor. However, sensitivity refers to how well the biosensor electrode detects an analyte without interference, while specificity refers to how well the biosensor electrode detects an analyte without any false positives. While it is simple to fabricate and miniaturize biosensors, different study results show that biosensors fabricated with a biopolymer have lower specificity than those manufactured with a semiconductor [41]. Additionally, these sensors applied to the surface of biomolecular recognition elements can significantly damage their binding ability. Therefore the ideal biosensor would have to be accurate, sensitive, and label-free, with the capability of direct pathogen detection.

However, a fundamental concern in biosensing is finding ways to measure biomolecules without interference from other molecules or cells. This is challenging because they are often present at low concentrations, making it difficult to measure them using conventional methods. Typical biosensors need to be more accurate to provide a high-quality reading. They also require more modification to work with the platform. The accuracy is nontrivial, and the elements necessary for recognition are not trivial either [42,43].

The study was performed on three different electrode designs to evaluate the possibility of identifying Gram-negative bacteria without incorporating biorecognition elements by measuring a solution’s cyclic voltammetry (CV). It is an innovative way to detect these bacteria with a high degree of accuracy, making it easier for scientists and clinicians to identify them.

The bacterial cells are surrounded by a multilayered, complicated structure that protects the organisms from aggressive conditions. The Gram-negative bacteria are covered by a cell wall composed of peptidoglycan and lipopolysaccharides. The outer membrane is made of lipopolysaccharides, which are in contact with the extracellular space [44].

Low-voltage electricity applied to the bacterial cell for minutes will build up negative charges on the cell surface and disrupt the integrity of cell membranes leading to a practical way to kill bacteria. This is due to the leaky membranes of these cells. Therefore, a low voltage is applied to the cell membrane of *E. coli* can create holes in the cell membrane and cause leakage of ions and proteins, which may lead to the death of bacteria [45,46,47,48]. Therefore, detecting these compounds can be a key to developing fast and rapid label-free biosensors without the complex steps of incorporating a biorecognition element.

**Figure 2:**
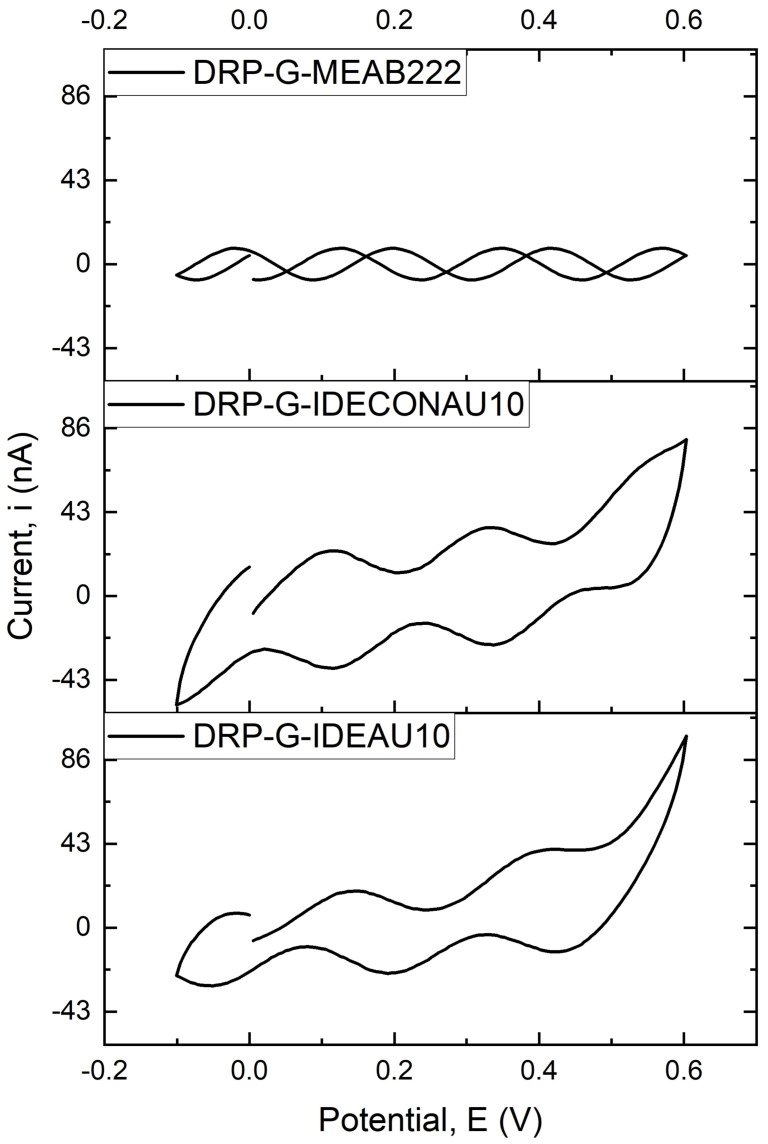
Cyclic voltammogram (CV) of three different electrodes using analyte solution.

Figure 2 demonstrates the cyclic voltammogram (CV) conducted on three different gold electrodes using an analyte consisting of normal saline (as cell suspension) solution and the whole bacterial culture with the change in cathodic and anodic current in the voltage range from −0.1 to +0.6 V at a scan rate of 100 mV/s. It is noted that the area of the CV curve of the G-IDECONAU10 electrode is much bigger than that of the other two electrodes, which indicates that the sensor is more susceptible to the solution that contains the bacterial cells. Consequently, the peak current is higher than the other two electrodes. According to these observations, the G-IDECONAU10 electrode is the more sensitive design to the bacterial cells and was selected to proceed further in the current study. Therefore, the concentric electrodes can detect and respond to biological substances and produce a signal that an electrode can easily detect. In addition, the concentric electrodes are much larger than the surface area of the parallel electrodes, which leads to lower losses and a higher Q factor in the concentric electrodes.

**Figure 3:**
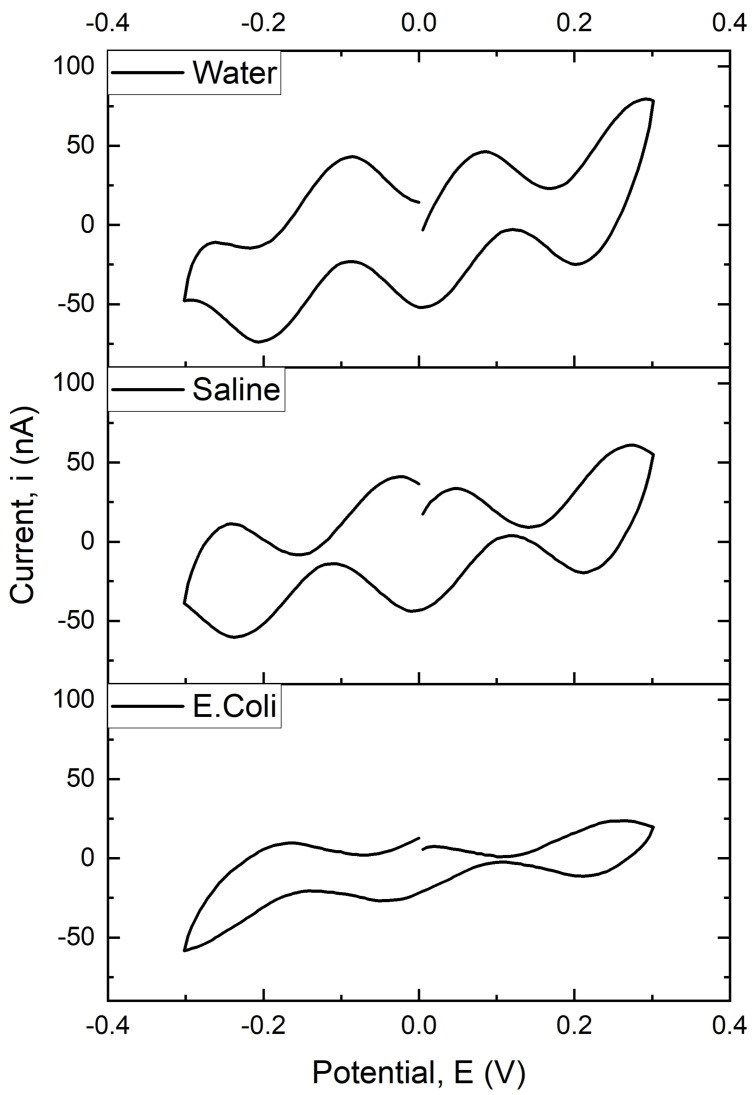
Cyclic voltammogram (CV) of G-IDECONAU10 electrode using three different media.

Figure 3 shows the electrochemical behavior of different electroactive for water (pH = 7), normal saline solution, and *E. coli* from the use of a G-IDECONAU10 electrode. Furthermore, the cyclic voltammogram curves show an apparent variation in peak current. The current peaks correspond to the oxidation and reduction reactions at the electrode surface. The results were obtained from a cyclic voltammogram with a particular focus on two discrete (localized) redox centers that appeared in all three electrode samples, indicating multiple electron transfer reactions, as well as determining the kinetic parameters for redox reactions at an electrode surface and the diffusion coefficient of various ions in solution. The result explains how quickly electrons react at an electrode surface and what reaction occurs.

The electrochemical process at the working electrode’s surface is mainly governed by diffusion. This is because the surface of an electrode is always in contact with a liquid medium so that it can be quickly depleted of charge carriers. The surface will then become more positive, allowing a greater flux of ions through to the electrode and re-establish equilibrium. This electrochemical process might be controlled by Nerstian diffusion by surface charge, and it is a reversible process that occurs at the surface electrode. This experiment’s results show that low scan rates measure fast reactions, while high scan rates are used for slower reactions. Consequently, the electrochemical process was thermodynamically controlled at a low scan rate, whereas, at a high scan rate, the process was kinetically controlled [49,50,51]. These results indicate how diffusion rates determine the rate of reactions in an electrochemical process. It also reveals that at higher scan rates, the diffusion rate is more than the rate of reaction because the diffusion rate is the rate at which molecules or ions pass through a given area, while the rate of reaction is the speed at which chemical reactions occur.

**Figure 4:**
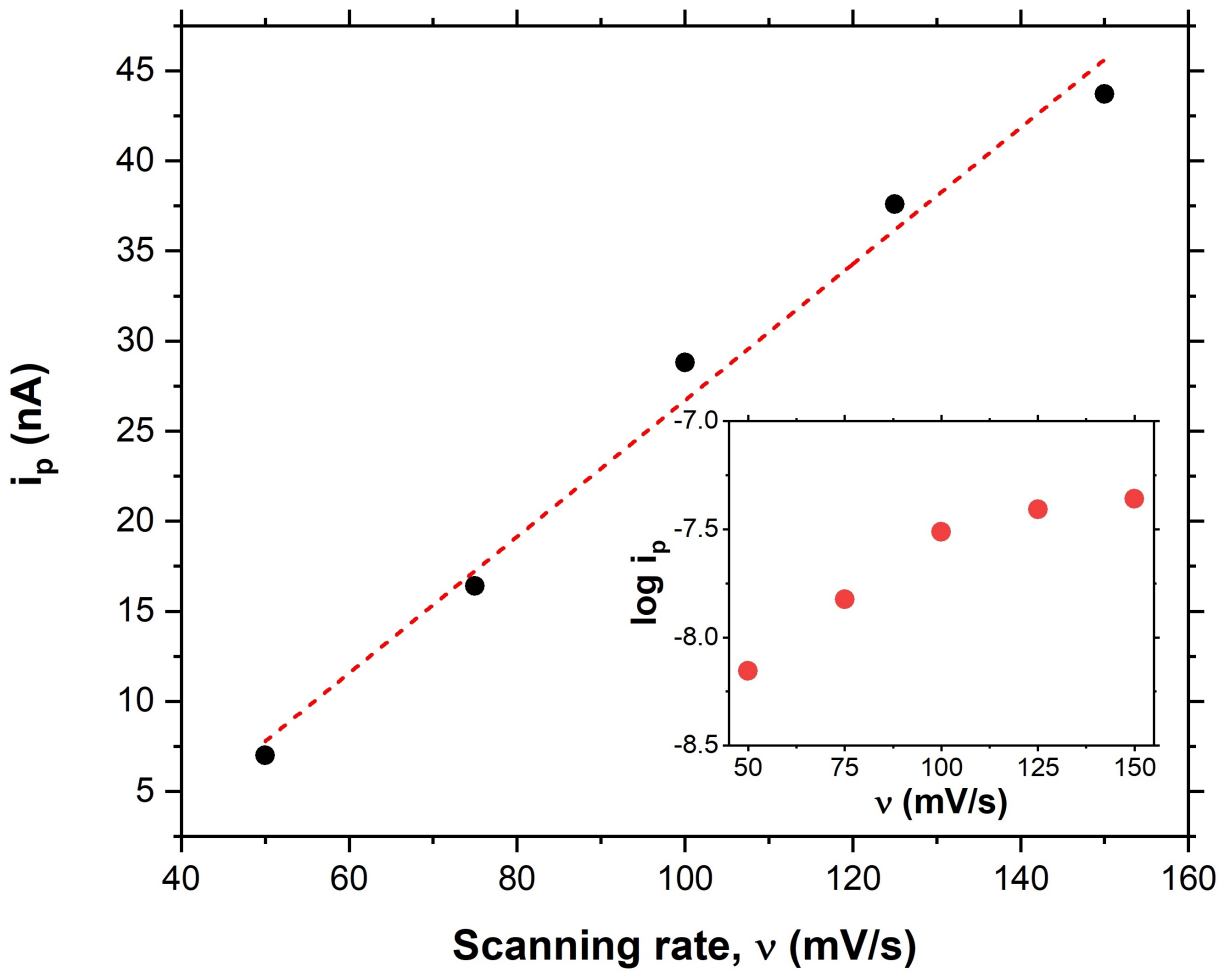
The linear regression of peak current at different scanning rates. Inset: the logarithmic behavior of peak current at different scanning rates.

Therefore, when the current at a higher scan rate increases, the charging current increases as the scan rate increases. The Faradaic current is proportional to the charge density and the scan rate. The passing of Faradaic currents through the electrode is directly proportional to the concentration change at the electrode surface. Accordingly, the higher scan rate will increase the slope since the ionic diffusion is constant. This will result in a higher current effect of scan rate on the peak current, as described in the Randles–Sevcik equation, directly proportional to the square root of the scan rate [52,53]. Furthermore, experiments showed that the peak current increases as we increase the scan rate, which means that the electrochemical reactions are kinetically controlled (Figure 4).

To determine the relationship between the observed CV peaks and the cell surface or spent media, we needed to account for the varying degrees of analyte concentration. The CV measurements were then analyzed using a linear regression model to fit for a relationship between CV peak height and analyte concentration. The CV scan rates were as follows: 50, 75, 100, 125, and 150 mV/s. Next, peak current was measured and plotted versus scan rate in Figure 4. For this examination, a linear correlation of peak current to scan rate revealed that the peak is correlated to the cell surface. In contrast, a logarithmic fit (inset Figure 4) shows that the peak is correlated to the spent media [54].

**Figure 5:**
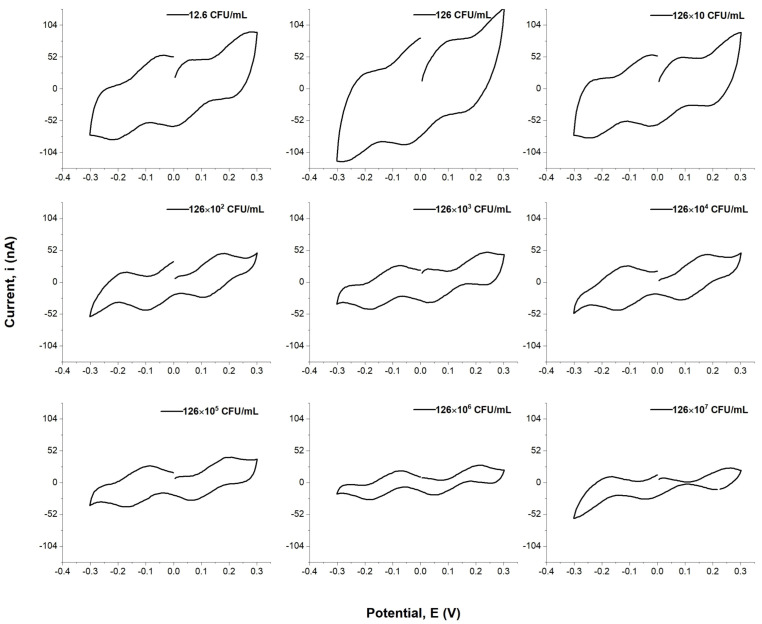
Cyclic voltammogram curves at different *E. coli* concentrations.

Figure 5 shows that it is clear that the cyclic voltammogram curves demonstrate that the peak current is related to the concentration of *E. coli*. The data obtained from these experiments can be used to calculate an appropriate concentration for a desired current value, which would then be used in a kinetic analysis or other investigation involving the electrochemical detection of *E. coli*. In addition, they could potentially lead to new insights into microbial behavior in various environments. The results indicate that the peak currents of the curves negatively correlate to the concentration of *E. coli* in solution, and current outputs decrease as concentrations increase. Furthermore, the figure revealed that as concentration decreases, the area of the CV curves increase, and the peak current increases as well.

Thus, let us describe how bacteria use these electric fields to their advantage. First, bacteria are coated with an electrical double layer and have a net negative charge on the surface of their membrane. Second, bacteria use electrostatic forces to create a positive or negative charge on their outer membrane, which causes them to polarize when they come into contact with a positive or negative electrode. This polarization causes bacterial adsorption, allowing bacteria to attach themselves to electrode surfaces more easily.

This can be explained as follows. When the bacteria concentration increases, negatively charged *E. coli* migrate to the electrode as the electric field increases. Since bacteria are negatively charged, they are attracted to positively charged electrodes and repelled by negative electrodes.

**Figure 6:**
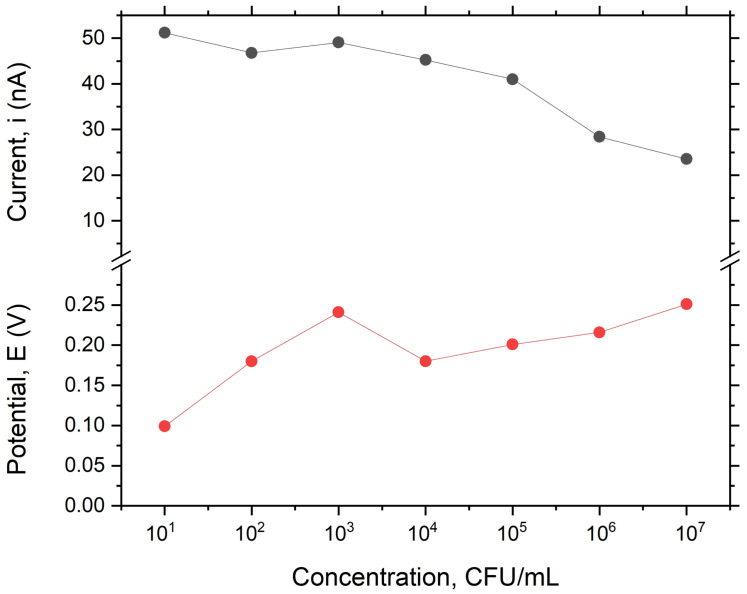
Variation in peak current and potential with *E. coli* concentration.

However, the electric field increases the number of bacteria that migrate to the electrode due to the higher gradient between the concentration of positively charged ions in the analyte solution, as well as the number of bacteria at the electrode surface, which causes more of these ions to diffuse from the analyte solution to the electrode surface. As these ions diffuse from the analyte solution into the electrode surface, they carry their positive charge along with them, thereby increasing their local concentration at this specific region and attracting more negatively charged bacteria. Furthermore, these bacterial cells can be captured at an electrode surface by applying a voltage pulse. The voltage is applied to electrodes and the bacteria, resulting in a steady-state current drop, and the maxim peak current of the analyte decreases almost linearly with the concentration of *E. coli* in the analyte solution. Therefore, the cyclic voltammetry has the lowest detectable concentration at 10${}^{2}$ CFU/mL, which depends on the number of bacterial cells in the analyte solution. However, we found that the quantification of concentration below 10${}^{2}$ CFU/mL was unreliable, which might be due to weak electroadsorption processes between the *E. coli* and the electrodes. (Figure 6). Nevertheless, this technique makes false positives quite probable, as any bacterial cell with a comparable size and negative charge delivers a comparative signal.

## 4. Conclusions

This article discusses the potential of utilizing a bioreceptor-free concentric interdigitated microelectrode to identify label-free bacterial cells. We found that this method is inexpensive, fast, and robust. The electrode design geometry has been studied, and it was found to be an effective method for identifying these bacteria without using labels, expensive equipment, or time-consuming techniques. The qualitative electrochemical detection technique is a promising strategy for identifying and detecting *Escherichia coli*, resulting in detection limits of 10${}^{2}$ CFU/mL, an improvement of at least one order of magnitude compared to the current state of the art. Sensitivity and specificity are the two most essential factors in determining the quality of a test. The G-IDECONAU10 sensor design is a promising bioreceptor-free system that is more susceptible to living bacterial cells suspended in a normal saline solution. In addition, cyclic voltammetry will provide an inexpensive, rapid, and accurate method to differentiate between different groups of bacteria and requires approximately a few hours for presumptive positive/negative results. In addition, the concentric interdigitated microelectrodes have a much larger surface area than the parallel electrodes, leading to lower losses and a higher Q factor. Therefore, the more susceptible design of concentric interdigitated microelectrodes enables the detection of smaller bacterial cells with higher sensitivity and selectivity than ever before. Furthermore, due to the lack the bioreceptors, these sensors can be used for various applications, such as on-chip detection. Therefore, this type of sensor will be many forthcoming developments, including developing materials with robust mechanical properties, reproducibility, and reduced manufacturing cost.

## Data Availability

The data presented in this study are available on request from the corresponding author.

## References

[1] World Health Organization (2009). Cardiovascular Diseases (cvds). https://www.who.int/news-room/fact-sheets/detail/cardiovascular-diseases-(cvds).

[2] Braissant O., Astasov-Frauenhoffer M., Waltimo T., Bonkat G. (2020). A review of methods to determine viability, vitality, and metabolic rates in microbiology. Front. Microbiol..

[3] Ishiki K., Nguyen D.Q., Morishita A., Shiigi H., Nagaoka T. (2018). Electrochemical detection of viable bacterial cells using a tetrazolium salt. Anal. Chem..

[4] Yang Q., Olaifa K., Andrew F.P., Ajibade P.A., Ajunwa O.M., Marsili E. (2022). Assessment of physiological and electrochemical effects of a repurposed zinc dithiocarbamate complex on Acinetobacter baumannii biofilms. Sci. Rep..

[5] Pangajam A., Theyagarajan K., Dinakaran K. (2020). Highly sensitive electrochemical detection of E. coli O157: H7 using conductive carbon dot/ZnO nanorod/PANI composite electrode. Sens.-Bio-Sens. Res..

[6] Lee J., Tatsumi A., Abe K., Yoshida W., Sode K., Ikebukuro K. (2014). Electrochemical detection of pathogenic bacteria by using a glucose dehydrogenase fused zinc finger protein. Anal. Methods.

[7] Vu Q.K., Tran Q.H., Vu N.P., Anh T.L., Le Dang T.T., Matteo T., Nguyen T.H.H. (2021). A label-free electrochemical biosensor based on screen-printed electrodes modified with gold nanoparticles for quick detection of bacterial pathogens. Mater. Today Commun..

[8] Garcia D.E., Chen T.H., Wei F., Ho C.M. (2010). A parametric design study of an electrochemical sensor. JALA J. Assoc. Lab. Autom..

[9] Amiri M., Bezaatpour A., Jafari H., Boukherroub R., Szunerits S. (2018). Electrochemical methodologies for the detection of pathogens. ACS Sens..

[10] Cesewski E., Johnson B.N. (2020). Electrochemical biosensors for pathogen detection. Biosens. Bioelectron..

[11] Halford C., Gau V., Churchill B.M., Haake D.A. (2013). Bacterial detection & identification using electrochemical sensors. JoVE (J. Vis. Exp.).

[12] Li Y., Fan P., Zhou S., Zhang L. (2017). Loop-mediated isothermal amplification (LAMP): A novel rapid detection platform for pathogens. Microb. Pathog..

[13] Velusamy V., Arshak K., Korostynska O., Oliwa K., Adley C. (2010). An overview of foodborne pathogen detection: In the perspective of biosensors. Biotechnol. Adv..

[14] Chambers J.P., Arulanandam B.P., Matta L.L., Weis A., Valdes J.J. (2008). Biosensor recognition elements. Curr. Issues Mol. Biol..

[15] Umesha S., Manukumar H. (2018). Advanced molecular diagnostic techniques for detection of food-borne pathogens: Current applications and future challenges. Crit. Rev. Food Sci. Nutr..

[16] Simoska O., Stevenson K.J. (2022). Electrochemical sensors for detection of Pseudomonas aeruginosa virulence biomarkers: Principles of design and characterization. Sens. Actuators Rep..

[17] Castle L.M., Schuh D.A., Reynolds E.E., Furst A.L. (2021). Electrochemical sensors to detect bacterial foodborne pathogens. ACS Sens..

[18] Karbelkar A.A., Furst A.L. (2020). Electrochemical diagnostics for bacterial infectious diseases. ACS Infect. Dis..

[19] Wang Y., Ma X., Qiao X., Yang P., Sheng Q., Zhou M., Yue T. (2021). Perspectives for Recognition and Rapid Detection of Foodborne Pathogenic Bacteria Based on Electrochemical Sensors. eFood.

[20] Kuss S., Amin H.M., Compton R.G. (2018). Electrochemical detection of pathogenic bacteria—Recent strategies, advances and challenges. Chem.–An Asian J..

[21] Conteduca D., Brunetti G., Dell’Olio F., Armenise M.N., Krauss T.F., Ciminelli C. (2019). Monitoring of individual bacteria using electro-photonic traps. Biomed. Opt. Express.

[22] Wolfbeis O.S. (2006). Fiber-optic chemical sensors and biosensors. Anal. Chem..

[23] Hoa X.D., Kirk A., Tabrizian M. (2007). Towards integrated and sensitive surface plasmon resonance biosensors: A review of recent progress. Biosens. Bioelectron..

[24] Dye C. (2014). After 2015: Infectious diseases in a new era of health and development. Philos. Trans. R. Soc. B Biol. Sci..

[25] Alahi M.E.E., Mukhopadhyay S.C. (2017). Detection methodologies for pathogen and toxins: A review. Sensors.

[26] Purohit B., Vernekar P.R., Shetti N.P., Chandra P. (2020). Biosensor nanoengineering: Design, operation, and implementation for biomolecular analysis. Sens. Int..

[27] McKeague M., DeRosa M.C. (2012). Challenges and opportunities for small molecule aptamer development. J. Nucleic Acids.

[28] A. Alonso-Lomillo M., Dominguez-Renedo O. (2017). Screen-printed biosensors in drug analysis. Curr. Pharm. Anal..

[29] Gopal A., Yan L., Kashif S., Munshi T., Roy V.A., Voelcker N.H., Chen X. (2022). Biosensors and Point-of-Care Devices for Bacterial Detection: Rapid Diagnostics Informing Antibiotic Therapy. Adv. Healthc. Mater..

[30] Suhito I.R., Koo K.M., Kim T.H. (2020). Recent advances in electrochemical sensors for the detection of biomolecules and whole cells. Biomedicines.

[31] Mahshid S.S., Dabdoub A. (2020). Development of a novel electrochemical immuno-biosensor for circulating biomarkers of the inner ear. Biosens. Bioelectron..

[32] Munteanu F.D., Titoiu A.M., Marty J.L., Vasilescu A. (2018). Detection of antibiotics and evaluation of antibacterial activity with screen-printed electrodes. Sensors.

[33] Lei K.F., Wu M.H., Hsu C.W., Chen Y.D. (2014). Real-time and non-invasive impedimetric monitoring of cell proliferation and chemosensitivity in a perfusion 3D cell culture microfluidic chip. Biosens. Bioelectron..

[34] Lin Y.K., Wu H.J., Hieu N.V., Chu P.Y., Do T.V.T., Yao F.Y.D., Phan T.L., Ching C.T.S. (2022). A New Biorecognition-Element-Free ID*μ*E Sensor for the Identification and Quantification of E. coli. Biosensors.

[35] Schackart K.E., Yoon J.Y. (2021). Machine learning enhances the performance of bioreceptor-free biosensors. Sensors.

[36] Therisod R., Tardif M., Marcoux P.R., Picard E., Jager J.B., Hadji E., Peyrade D., Houdré R. (2018). Gram-type differentiation of bacteria with 2D hollow photonic crystal cavities. Appl. Phys. Lett..

[37] Ahmed A., Rushworth J.V., Hirst N.A., Millner P.A. (2014). Biosensors for whole-cell bacterial detection. Clin. Microbiol. Rev..

[38] Band Microelectrodes Array Ref. G-MEAB222—Metrohm DropSens. https://www.metrohm.com/en/products/g/-mea/g-meab222.html.

[39] InterDigitated Concentric Gold Electrodes Ref. G-IDECONAU10—Metrohm DropSens. https://www.metrohm.com/en/products/g/-ide/g-ideconau10.html.

[40] InterDigitated Gold Electrodes Ref. G-IDEAU10–Metrohm DropSens. https://www.metrohm.com/en/products/g/-ide/g-ideau10.html.

[41] Liu Y., Hao M., Chen Z., Liu L., Liu Y., Yang W., Ramakrishna S. (2020). A review on recent advances in application of electrospun nanofiber materials as biosensors. Curr. Opin. Biomed. Eng..

[42] Welch N.G., Scoble J.A., Muir B.W., Pigram P.J. (2017). Orientation and characterization of immobilized antibodies for improved immunoassays. Biointerphases.

[43] Cimafonte M., Fulgione A., Gaglione R., Papaianni M., Capparelli R., Arciello A., Bolletti Censi S., Borriello G., Velotta R., Della Ventura B. (2020). Screen printed based impedimetric immunosensor for rapid detection of Escherichia coli in drinking water. Sensors.

[44] Silhavy T.J., Kahne D., Walker S. (2010). The bacterial cell envelope. Cold Spring Harb. Perspect. Biol..

[45] Asadi M.R., Torkaman G. (2014). Bacterial inhibition by electrical stimulation. Adv. Wound Care.

[46] Hahn E., Wild P., Hermanns U., Sebbel P., Glockshuber R., Häner M., Taschner N., Burkhard P., Aebi U., Müller S.A. (2002). Exploring the 3D molecular architecture of Escherichia coli type 1 pili. J. Mol. Biol..

[47] Krishnamurthi V.R., Rogers A., Peifer J., Niyonshuti I.I., Chen J., Wang Y. (2020). Microampere electric current causes bacterial membrane damage and two-way leakage in a short period of time. Appl. Environ. Microbiol..

[48] Vollmer W., Blanot D., De Pedro M.A. (2008). Peptidoglycan structure and architecture. FEMS Microbiol. Rev..

[49] Nicholson R.S. (1966). Semiempirical Procedure for Measuring with Stationary Electrode Polarography Rates of Chemical Reactions Involving the Product of Electron Transfer. Anal. Chem..

[50] Lee K.J., Elgrishi N., Kandemir B., Dempsey J.L. (2017). Electrochemical and spectroscopic methods for evaluating molecular electrocatalysts. Nat. Rev. Chem..

[51] Compton R.G., Perkin S.J., Gamblin D.P., Davis J., Marken F., Padden A.N., John P. (2000). Clostridium isatidis colonised carbon electrodes: Voltammetric evidence for direct solid state redox processes. New J. Chem..

[52] Randles J.E. (1948). A cathode ray polarograph. Part II.—The current-voltage curves. Trans. Faraday Soc..

[53] Sevcik A. (1948). scillographic Polarography with Periodical Triangular Voltage. Collect. Czechoslov. Chem. Commun..

[54] Laviron E. (1983). Electrochemical reactions with protonations at equilibrium: Part VIII. The 2 e, 2H^+^ reaction (nine-member square scheme) for a surface or for a heterogeneous reaction in the absence of disproportionation and dimerization reactions. J. Electroanal. Chem. Interfacial Electrochem..

